# Determinants of Parents Taking Their Children for Scheduled Vaccinations during COVID-19 Pandemic in South Africa

**DOI:** 10.3390/vaccines11020389

**Published:** 2023-02-08

**Authors:** Mokhantso Makoae, Tholang Mokhele, Inbarani Naidoo, Sibusiso Sifunda, Ronel Sewpaul

**Affiliations:** 1 Developmental, Capable and Ethical State Division, Human Sciences Research Council, Cape Town 8000, South Africa; 2 eResearch Knowledge Centre, Human Sciences Research Council, Pretoria 0001, South Africa; 3 Human and Social Capabilities Division, Human Sciences Research Council, Durban 4001, South Africa; 4 Human and Social Capabilities Division, Human Sciences Research Council, Pretoria 0001, South Africa; 5 Human and Social Capabilities Division, Human Sciences Research Council, Cape Town 8000, South Africa

**Keywords:** South Africa, parental views, scheduled vaccinations, COVID-19, preventable childhood diseases

## Abstract

Scheduled or routine childhood vaccinations are known for their effectiveness in eradicating fear for many life-threatening and disabling diseases and saving lives globally. This paper is aimed at assessing determinants of parents taking their children for scheduled vaccinations during the COVID-19 pandemic in South Africa. Data used for this paper were obtained from the Human Sciences Research Council’s (HSRC) COVID-19 Online Survey titled “One Year Later Survey”, which was conducted between 25 June and 11 October 2021 in South Africa. Multivariate logistic regression analysis was performed to achieve this study goal. Findings showed that just over half of parents (56.7%) reported taking their children for scheduled vaccinations across the country. Males were significantly less likely (aOR = 0.53 95% CI [0.45–0.61], *p* < 0.001) to have taken their children for scheduled vaccinations than females. Parents’ experiences and views were among key determinants of parents having taken their children for scheduled vaccinations in South Africa. Parents who had never taken influenza (flu) vaccines were significantly less likely (aOR = 0.33 [0.28–039], *p* < 0.001) to have taken their children for scheduled vaccinations than those who had taken flu vaccines. Parents who did not know anyone who had personally experienced serious side effects to any vaccine were significantly less likely (aOR = 0.77 [0.66–0.90], *p* = 0.001) to have taken their children for scheduled vaccinations than those who knew anyone who had experienced them. Parents who did not think vaccines were a good way to protect communities from disease were significantly less likely (aOR = 0.50 [0.33–0.77], *p* = 0.001) to have taken their children for scheduled vaccinations than those who thought vaccines were a good way to protect communities from disease. These findings are of significance especially during the time when the country is still struggling to reach a substantial proportion of its population vaccinated for COVID-19. Thus, these findings may be relevant in determining parents’ intentions to have their children receive the South African Department of Health recommended vaccines for their respective age group.

## 1. Introduction

Scheduled or routine childhood vaccines are known for their effectiveness in eradicating fear of many life-threatening and disabling diseases and saving lives globally, and several governments have institutionalised childhood immunization programmes. However, during disasters such as COVID-19 pandemic, immunisation uptake can be disrupted. The World Health Organization (WHO) reported that 25 million children missed out on vaccination in 2021 due to COVID-19 disruptions to health care systems. COVID-19 lockdowns disrupted routine childhood vaccine coverage for under-five-year-olds globally [1], leading to significant declines in vaccination rates. Disruptions are creating a risk for infectious disease outbreaks, but the extent to which these possible outbreaks are a source of concern to parents may be unclear. Average periodic records of routine vaccines and proportions of children with up-to-date vaccination for age decreased in the early stages of the pandemic [2]. 

Parents’ decision making about initiating or continuing their children’s scheduled immunisation is compounded by contextual changes and psychological factors that emerge during crises. Delayed immunisation and nonvaccination compromise protection of children from outbreaks of vaccine-preventable diseases and death [3]. South Africa has a relatively long history with its routine childhood vaccination programme, which started in 1974 [4], though not comparable to some developed countries such as the United Kingdom’s availability of viral smallpox vaccination, which began in the 18th century [5] and was later followed by development and bacterial vaccination for tuberculosis. South Africa has since introduced a suite of routine childhood vaccines that form part of the national expanded immunisation programme (EIP-SA) funded by the government. The childhood immunisation schedule entails vaccines administered at birth and subsequent doses until 12 years provided as follows: Bacillus Calmette-Guérin (BCG) and Oral polio vaccine (OPV)(0) at birth; OPV(1), Rotavirus (RV)(1), Diphtheria/Tetanus/Pertussis, inactive polio vaccine, Haemophilus influenzae and Hepatitis B antigens (DTP-IPV-Hib-HepB)(1) and Pneumococcal conjugate vaccine (PCV)(1) at six weeks; DTP-IPV-HiB-HepB(2) at 10 weeks; RV(2), DTP-IPV-Hib-HepB(3) and PCV(2) at 14 weeks; Measles(1) at six months; PCV(3) at nine months; Measles(2) at 12 months; DTP-IPV-Hib-HepB(4) at 18 months; and Tetanus-Diphtheria (Td)(1) at six years, followed by two doses of Human papilloma virus vaccine (HPV) at nine years and Td(2) at 12 years [6]. Although they are a crucial element of the universal health coverage, unlike in countries such as Saudi Arabia where completeness of preschool age immunization is mandatory [7], in South Africa childhood vaccines are not mandatory. 

Scheduled or routine childhood vaccines in South Africa are available for free in public primary health care facilities and are primarily offered for young children under the age of five years. The majority of them are to be taken in the first year of life. However, the global challenge of incomplete or no vaccination [8], also common in South Africa, means that the national government and provincial health authorities can prevent diseases that cause childhood suffering, disability, and death by improving the readiness of the health system to maintain optimal childhood vaccination rates even during disasters such as pandemics. During disasters in low- and middle-income countries, health care systems are severely disrupted thus exposing children to missing scheduled immunizations. A study conducted in India before COVID-19 reported about 18% decline in age-appropriate full immunization of children in disaster-affected areas [9]. 

Despite the proven benefits of childhood vaccines in eradicating life-threatening and disabling diseases, South Africa’s vaccine coverage in the pre-COVID-19 period was already suboptimal. Results from district health administrative data showed that national immunization coverage was 77% in 2017/2018 [10] (District Health Information System estimates do not accurately capture vaccinations administered in the private sector (Makamba-Mutevedzi et al., 2020). In 2019, Makamba-Mutevedzi and colleagues [11] reported a similar national average, 76.8% (75.4–78.2), from the nationally representative EPI survey of full vaccination coverage, that is, children who received all age-appropriate vaccinations from birth to 18 months. Disparities have been noted with some provinces and districts performing above national average coverage of routine immunisation and a handful of districts registering far below the national average [3,11]. Vaccine uptake determines full immunisation coverage, and vaccine timeliness is necessary for optimal prevention of debilitating childhood diseases and child death. 

Research has identified various factors contributing to childhood immunisation incompleteness, and some studies and evidence reviews identify parent-related reasons. The determinants of parental decision making about vaccination are categorised as religious reasons, personal beliefs, safety concerns, and desire to have information from the health care professional [12,13,14]. Evidence reviews identify parents’ low trust in a health system due to social exclusion [12] and lack of confidence in the safety and effectiveness of vaccines as some of the factors that lead to suboptimal immunisation rates [15]. A study conducted in South Africa on EPI managers’ perspectives on the challenges affecting immunisation coverage before the onset of COVID-19 reported that apart from systemic barriers that constrain the health system in supporting families in ensuring optimal immunisation, parents’ reluctance to take their children for immunisation also lowered national coverage [16]. A national EPI survey found that in South Africa parents refused some of the scheduled vaccines (BCG and OPV0), and some reported forgetfulness, but religious reasons were insignificant [11]. 

Understanding determinants of parental reasons for their children receiving, delaying, or not receiving routine vaccination in countries with high infection rates during COVID-19 outbreaks is crucial. A few reviews and studies have examined parental views and experiences about routine childhood vaccines and the challenges of routine immunization during disasters and pandemics such as COVID-19. A systematic review of how delivery of maternal and child health services is affected during public health emergencies and pandemics in low- and middle-income countries identified common challenges that studies report to include disruptions in public health care systems, immunization becoming irregular, many children missing routine vaccinations as scheduled, and postponing until the situation was normal to complete their routine immunization schedule [17]. It is crucial to understand the social and behavioural drivers of parental attitudes towards vaccines and routine childhood vaccine behaviour in a low- and middle-income country given the limited evidence on strategies that can improve parents’ attitudes towards routine immunization during COVID-19 outbreaks. 

Parental views and experiences are crucial because parents are an important group in society since they are not only responsible for their own health but also proxy for the health of their children [18,19]. They do not only participate in decision making about their children’s vaccination through consenting, but they also weigh the benefits of vaccinating or not vaccinating children during raging pandemics. Understanding parents’ views and experiences with vaccines can inform strategies needed to encourage parents to comply with the EPI programme and retain their children on scheduled vaccination programmes during pandemics such as COVID-19. This paper is aimed at assessing determinants of parents taking their children for scheduled vaccinations during the COVID-19 pandemic in South Africa.

## 2. Materials and Methods

### 2.1. Study Design and Sampling

Data used for this paper were obtained from the Human Sciences Research Council’s (HSRC) COVID-19 Online Survey titled “One Year Later Survey”, which was conducted between 25 June and 11 October 2021 in South Africa. The One Year Later Survey was conducted to assess the social and behavioural factors related to the pandemic, including vaccine-related attitudes, intentions, and behaviours. An online survey method, which was supplemented by telephonic interviews, was employed since face-to-face interviews were a challenge due to lockdown restrictions. Online surveys are more of convenience sampling as those with access to smart phones and internet are more likely to participate. To reduce this bias, telephonic interviews were included for disadvantaged areas such as townships and informal settlements. 

### 2.2. Target Population and Sampling Frame

The study population was all adults aged 18 years and older who resided in South Africa regardless of their population group, sex, religion, and nationality. In terms of exclusion criteria, respondents who reported that they did not have children were excluded from the study sample as the focus was on parents. There were no exclusion criteria set for age of children in this study.

### 2.3. Sample Size

There was no sample size calculation; thus, a targeted minimum sample size was not predetermined for this online survey. The realised sample size of 12,708 parents is regarded large enough for the purpose of this study. It has been shown that reweighted online samples can produce response patterns that are statistically similar to general population characteristics [20]. Therefore, the data from the survey were weighted to distribution of South Africa’s estimated parent population using the general population demographics by age, sex, population group, and province.

### 2.4. Study Procedures

Invitations to participate in the study were widely distributed on social media platforms, the HSRC website, radio, and television stations. These media platforms included WhatsApp, Facebook, Twitter, and Instagram. The survey was administered online using a data-free platform, and data collection was supplemented by telephonic interviews. In addition to being a data-free platform, Moya Messaging platform has a large user base of four million members and one million daily engaged users. Telephonic interviews were to ensure that population from disadvantaged areas such as townships and informal settlements who did not have access to smart phones and computers were not excluded. Both online and telephonic surveys were conducted in six of the 11 official languages of South Africa, namely English, Afrikaans, IsiZulu, IsiXhosa, Xitsonga, and Tshivenda.

### 2.5. Study Instrument

The questionnaire was developed based on the initial questionnaires of HSRC COVID-19 Online Surveys [21,22,23], which were primarily based on previous work on public reactions to the pandemic [24,25] and in consultation with socio-behavioural scientists, public health experts, and epidemiologists both locally and globally.

### 2.6. Measures

The primary outcome variable was respondents having taken their children for scheduled vaccinations. The following question was asked, “Have you taken your children for scheduled vaccinations?” with response being 1 = yes, 2 = no, and 3 = I don’t have children. These responses were further recoded into 1 = yes and 0 = no. Those who reported that they did not have children were excluded from the study sample as the focus was on parents. The explanatory variables included sociodemographic variables and variables that indicated parents’ experiences and views about vaccines in general (Table 1).

### 2.7. Data Analysis

Data analysis was conducted using Stata version 15 [26]. Data from the survey were benchmarked using the general population demographics by age, sex, population group, and province, based on Statistics South Africa’s 2021 population mid-year estimates [27]. The Stata “svy” command was used to incorporate benchmarking weights into the analysis. Descriptive analysis (frequencies and percentages) was used to summarize the sample characteristics of the study across demographic variables. Differences in the percentage of respondents who took their children for scheduled vaccinations versus those that did not were compared across the explanatory variables using 95% Confidence Intervals (CIs) and the Chi-square test. ArcGIS10.8 was used for map production [28].

Logistic regression models were considered as they are most appropriate statistical method for binary (yes or no) outcome variable [29,30]. One of the assumptions underlying the logistic regression models is that the explanatory variables should not be too highly correlated with each other, thus there should be little or no multicollinearity among the explanatory variables. Therefore, a correlation matrix was conducted to assess multicollinearity between explanatory variables. Estimates from logistic regression models, including multiple or multivariate logistic regression, which frequently used the multivariate technique, calculates odds ratios (ORs) and not Risk Ratios [30,31]. Some of the advantages of ORs are that they provide an estimate for the relationship between two binary variables, and they enable examining the effects of other variables on that relationship [32]. This means that the relationship between each variable and the binary outcome can be studied while holding constant the values of the other explanatory variables [28]. This is also useful to adjust the estimates for the effects of confounding variables in observational data [29]. Bivariate logistic regression models were conducted, and all significant variables were fitted into a multivariate logistic regression. Multivariate logistic regression analysis was performed to determine factors associated with parents taking their children for scheduled vaccinations during the COVID-19 pandemic in South Africa. Adjusted Odds Ratios (aOR) with 95% Confidence Intervals (CIs) were reported, and *p* value equal to or less than 0.05 was considered statistically significant.

## 3. Results

### 3.1. Characteristics of Study Sample

The study sample was 12,708 parents who responded to the question of having taken their children for scheduled vaccinations (Table 2). Female respondents or mothers accounted for 60.7% of the study sample. The majority of parents were Black Africans (80.6%), 42.6% had matric as their highest education qualification, 67.7% were unemployed, and 44.5% were residing in townships.

Regarding vaccine experiences, less than one third (30.5%) of the parents reported that they had taken the flu vaccine themselves. More than one in seven (15.2%) of the parents had personally refused to take any vaccine while over one quarter (26.3%) indicated that they have objected to allow someone else to take a vaccine. Parents who did not know anyone who had personally experienced serious side effects to any vaccine constituted 31.1%.

Respondents were asked questions about their views and opinions regarding vaccines in general. Less than two thirds (62.0%) believed that vaccines were a good way to protect communities from disease while more than half of (52%) the parents thought vaccines strengthen the immune system. The majority (72.4%) of the parents indicated that they would take the COVID-19 vaccines once they were made available while 10.3% reported that they would not take them.

### 3.2. Parents who Took Their Children for Scheduled Vaccinations by Sociodemographics, Experiences, and Views

Of the 12,708 parents, 56.7% reported that they had taken their children for scheduled vaccinations (Table 3). The proportion of parents who took their children for scheduled vaccinations differed significantly by sex, population group, age group, education level, employment status, and locality (*p* < 0.001). For instance, a significantly higher proportion of females (49.6%, 95% CI [47.6–51.6]) reported that they took their children for scheduled vaccinations compared to males (35.9%, [33.6–38.3]). Parents from rural or traditional tribal areas reported the lowest proportion of (34.8%, [31.5–38.2]) taking their children for scheduled vaccinations.

When considering parents’ experiences with vaccines, parents who had taken the flu vaccine had significantly higher percentage of (62.1%, [59.0–65.0]) having taken their children for scheduled vaccinations than those who had never taken flu vaccine. Those who had personally refused to take any vaccine had significantly higher percentage of (52.3%, [48.3–56.3]) having taken their children for scheduled vaccinations than those who had never done so. Parents who knew anyone who had personally experienced serious side effects to any vaccine had significantly higher proportion of (47.9%, [45.1–50.6]) having taken their children for scheduled vaccinations than those who did not know anyone.

In terms of parents’ views on vaccines, parents who thought vaccines are a good way to protect communities from disease had significantly higher proportion of (47.2%, [45.1–49.2]) having taken their children for scheduled vaccinations than those who did not think so and those who were not sure. Those who thought vaccines strengthen the immune system had significantly higher percentage of (48.4%, [46.2–50.5]) having taken their children for scheduled vaccinations than those who did not think so and those who were not sure.

**Figure 1:**
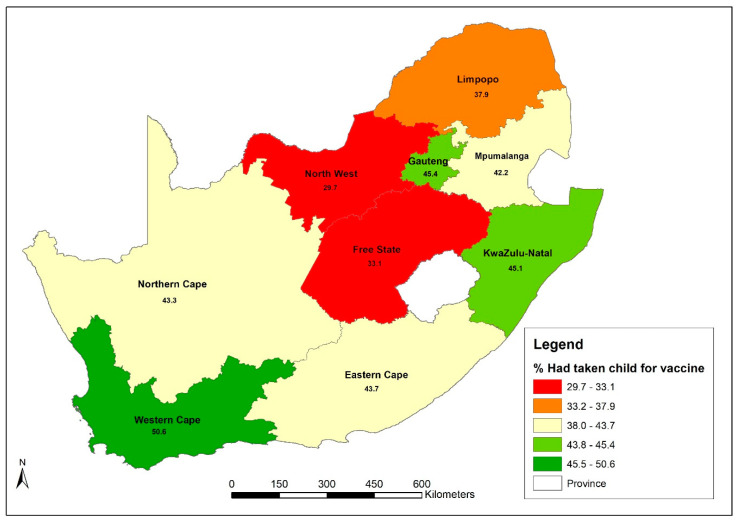
Map showing proportions of parents who took their children for scheduled vaccinations.

There was a significant difference (*p* < 0.001) in the proportions of parents who took their children for scheduled vaccinations by province. Western Cape had the highest proportion with 50.6% of parents who indicated that they had taken their children for scheduled vaccinations, followed by Gauteng and KwaZulu-Natal with 45.4% and 45.1%, respectively (Figure 1). Free State and North West provinces accounted for the lowest proportions with 33.1% and 29.7%, respectively.

### 3.3. Multivariate Logistic Regression Analysis of Factors Influencing Parents’ Taking Children for Scheduled Vaccinations

Males were significantly less likely (aOR = 0.53 95% CI [0.45–0.61], *p* < 0.001) to have taken their children for scheduled vaccinations than females (Table 4). Parents from the all other population groups were significantly more likely (Whites: aOR = 4.08 [2.79–5.96], *p* < 0.001; Coloureds: aOR = 1.72 [1.42–2.08], *p* < 0.001; Indian/Asians: aOR = 3.50 [2.23–5.49], *p* < 0.001) to have ever taken their children for scheduled vaccinations than Black Africans. The parents’ likelihood of taking their children for scheduled vaccinations gradually increased with increasing parental age as older parents were significantly more likely (30 to 39 years: aOR = 1.19 [1.05–1.35], *p* = 0.007; 40 to 49 years: aOR = 1.27 [1.08–1.49], *p* = 0.004; 50 to 59 years: aOR = 1.29 [1.02–1.64], *p* = 0.035; 60 years and older: aOR = 1.90 [1.24–2.90], *p* = 0.003) to have taken their children for scheduled vaccinations than younger people (those aged 18 to 29 years). Parents residing in suburbs, townships, and informal settlements were significantly more likely (aOR = 1.78 [1.33–2.38], *p* < 0.001; aOR = 1.41 [1.09–1.82], *p* = 0.009; aOR = 1.45 [1.04–2.03], *p* = 0.029 respectively) to have taken their children for scheduled vaccinations than those residing in the city.

In terms of parents’ experiences with vaccines as determinant factors, those who had never taken flu vaccines were significantly less likely (aOR = 0.33 [0.28–039], *p* < 0.001) to have taken their children for scheduled vaccinations than those who had taken flu vaccines. Parents who did not know anyone who had personally experienced serious side effects to any vaccine were significantly less likely (aOR = 0.77 [0.66–0.90], *p* = 0.001) to have taken their children for scheduled vaccinations than those who knew anyone who had experienced them. Parents who did not think vaccines were a good way to protect communities from disease were significantly less likely (aOR = 0.50 [0.33–0.77], *p* = 0.001) to have taken their children for scheduled vaccinations than those who thought vaccines were a good way to protect communities from disease.

## 4. Discussion

This study sought to explore and understand the determinants of parents taking their children for scheduled childhood vaccination during the COVID-19 pandemic in South Africa. Vaccination of the paediatric population is a significant public health intervention even during disasters such as COVID-19. As He and colleagues [33] stated, it is critical for policy makers and health care professionals to have adequate knowledge about factors that influence routine childhood vaccine uptake including hesitancy to maintain paediatric vaccination rates and promote vaccine confidence during and after the COVID-19 pandemic. 

In this study, the proportion of parents who reported taking their children for scheduled vaccinations was far smaller than the proportions reported by previous research including the nationally representative South African EPI survey conducted in the period prior to the COVID-19 pandemic [11]. It is always crucial that children scheduled or routine vaccination rates do not decrease during public health disasters and pandemics because missed or delayed immunisation can expose children to serious illness, disability, or death. Unfortunately, childhood immunisation programmes were reported to have been negatively affected by the pandemic globally, and the numbers of children who missed immunisation increased raising concerns about outbreaks of vaccine-preventable diseases due to the impact of COVID-19 on global health systems [33,34,35,36]. 

Our study findings showed that 52% of the parents thought vaccines strengthen the immune system while more than 39% were uncertain. Parents could have been concerned about exposing themselves or their young children to coronavirus infection by visiting public health facilities during the lockdowns. Bell and colleagues [37] found that even in a study with more than 85% of parents and guardians who believed that it was vital to adhere to paediatric vaccination schedules, “…this was balanced against their concerns over vaccinating their children during the pandemic…participants discussed the weighing up of perceived risks and benefits of taking their children for vaccination” [37] (p. 12). The context in which vaccination takes place is as important as parents’ characteristics in influencing routine childhood vaccination. 

Our findings also showed that there were clear differences in parents’ gender and age regarding them reporting vaccinating their children during COVID-19. Fathers or male caregivers were less likely to have taken their children for scheduled vaccinations than mothers or female caregivers. This does not necessarily mean that fathers were against their children receiving scheduled vaccinations but is because mothers are the ones who mostly take responsibility for children’s caregiving and health; hence, they have a better chance of taking them for vaccinations. Notably, a review synthesis shows that especially in low- and middle-income countries, including in Africa, child vaccination facilities as social settings are highly feminised and can make men feel alienated from this social role [12]. If the women were fearful about risks associated with COVID-19, their behaviour would change despite the delivery of routine vaccination remaining unchanged, as indicated by Sahoo and colleagues: “…however, women often did not prefer to visit the immunization site, due to fear or suspicion of infection. Some studies reported perceptions about contracting the infection among children through injections in healthcare facilities” [12] (p. 9). Notably, in another study, they found that mothers were more fearful than fathers about risks associated with COVID-19 vaccination in Saudi Arabia [38].

Furthermore, in our analysis, younger parents (18 to 29 years old) were more hesitant to vaccinate their children than older parents. The parents’ likelihood of ever taking their children for scheduled vaccinations gradually increased with increasing parental age. With the onset of COVID-19, parents’ practices with maintaining scheduled vaccines and their appraisal of the benefits of vaccination during the pandemic would change because these decisions are hardly ever static [12]. They were influenced by how well informed the parents were with the national childhood vaccination programme. Young parents would not have as much experience with vaccines as older parents. One of the well-documented reasons for parents’ hesitancy to vaccinate their children is lack of information and their need to be provided with adequate information about the benefits and risks of vaccines under any circumstance, so they can make informed decisions [14]. Considering that health communication resources during the pandemic were mostly devoted to the developments in the response of government to the pandemic, reports of dwindling childhood vaccination rates could have been easily drowned by daily reports about South Africa’s pandemic. Young parents would have been disadvantaged by lack of information about whether to vaccinate children considering that their experience with vaccines would only be at embryonic stage compared with older parents who probably had longer history with these interventions. This means that they would tremendously benefit from clinic visits where health professionals consistently provided them with vaccination information. 

It is not plausible that the young parents in our sample would be similar to parents in high-income countries who actively seek information on childhood vaccines to decide based on their particular and individual child if they indeed will benefit from any specific vaccine [12]; young parents would rely on government decision makers and health professionals for vaccination information during time of uncertainty. Since parental lack of knowledge about childhood vaccines was found to be associated with younger age of parents, it is important for the Department of Health to intensify health literacy of young parents and consistently provide them with information on childhood immunization, vaccines, and technology used, as well as the benefits of immunization, to address possible uncertainty during periods of reduced social interaction at health facilities. This finding is paradoxical but crucial as South Africa may also be different from other middle-income countries where studies report that younger parents tend to have better knowledge about childhood immunization than the older age cohorts of parents [7]. 

Concerns about catching coronavirus while accessing health services are real and require effective communication by health authorities and professionals emphasising availability of these services during outbreaks and health education about the benefits of up-to-date scheduled childhood vaccines. Evidence-based health policy guidelines and communication can facilitate parental decision making about children’s immunization schedule during uncertainty. When parents discontinue or suspend contact with the vaccination facilities, the number of unvaccinated children increases. This situation is associated with outbreaks of vaccine-preventable diseases such as measles. Already in 2022, some of the provinces with lower than national immunization coverage have reported measles outbreaks threatening the health and survival of young children. 

Our findings also showed some racial differences whereby Black Africans reported the lowest levels of taking children for scheduled vaccinations when compared to other racial groups. This could be due to several factors such as access to health facilities, poverty, and unemployment as well as other racial disparities that are found in South African society. The findings of this study further showed that parents residing in suburbs, townships, and informal settlements were significantly more likely to have ever taken their children for scheduled vaccinations than those residing in the inner cities. It is important to note that although the term city is inclusive of informal settlements, townships, and suburbs, the sociodemographic characteristics of these areas are distinctly different given the history of extensive state interference in urban development in South Africa [39,40]. Parents who reside in most inner cities in South Africa are mostly young people who at the later stage move to surrounding suburbs and townships. Additionally, COVID-19 disproportionately occurred in urban areas, and government lockdowns accentuated the “second-order impacts” of the pandemic related to health service disruptions on the vulnerable urban populations [41]. This notion supports the finding that inner city residents were less likely to have ever taken their children for scheduled vaccinations as parents’ likelihood of ever taking their children for scheduled vaccinations has been found to be positively associated with increasing parental age in this study. 

Among the parents/caregivers who participated in our online survey and had opted to vaccinate their children in the past, about two fifths indicated they had objected to allowing someone else to take a vaccine, which is lower than the proportion who objected to taking the COVID-19 vaccination in other countries. For instance, 52%, (n = 1094) of parents were unsure or not intending to vaccinate their children in Australia [42] whilst 73% (n = 274) of parents expressed negative or indecisive views on vaccinating their children in Turkey [43]. 

Parents’ views or beliefs about the efficacy of childhood vaccines influence whether they had taken their children for scheduled vaccinations. Parents who believed vaccines offered no protection from disease were significantly less likely to have taken their children for scheduled vaccinations than those who thought vaccines were a good way to protect communities from disease suggesting that knowledge about vaccine efficacy might be a driver in decision making. When considering parents’ views on vaccines as determinants of having taken their children for scheduled vaccinations, only one out of three variables was found to be significant. That is, those who did not think vaccines were a good way to protect communities from disease were significantly less likely to have taken their children for scheduled vaccinations than those who thought vaccines were a good way to protect communities from disease. Certain health behaviours of parents in relation to own health were associated with their behaviour when it came to protecting their children’s health from vaccine-preventable diseases. Parents who had never taken flu vaccines were significantly less likely to have ever taken their children for scheduled vaccinations than those who had taken flu vaccines. Surprisingly, parents who did not know anyone who had personally experienced serious side effects to any vaccine were significantly less likely to have ever taken their children for scheduled vaccinations than those who knew someone who had experienced such. This finding suggests that knowledge about the risk of experiencing side effects of vaccines was not influential in the parents’ decision making. The finding could be pointing to the importance of parents’ knowledge about childhood vaccination and diseases that are targeted with these interventions, as Alshammari and team [7] found that good knowledge about childhood vaccines increased the likelihood of parental adherence to scheduled vaccines. The challenge, however, would be a seeming low participation of parents in the sample in other disease prevention interventions such as flu vaccination taken periodically. 

The study has some limitations. Firstly, the outcome variable is subject to self-report because a parent’s reporting of whether they took their child for their scheduled vaccinations does not fully measure vaccination status among their child/ren, and this also does not fully encompass parental experience of taking their children for scheduled vaccinations. Secondly, the survey was conducted as a general population survey administered online and telephonically, and thus the sample methodology was not aimed at specifically sampling parents of young children. Nevertheless, the study uses data from parents to assess patterns of associations between their experiences and views with vaccines and whether they reported taking their children for their scheduled vaccinations. In light of the COVID-19 vaccine hesitancy observed during the pandemic, the findings from this study may be relevant in determining parents’ intentions to have their children receive the South African Department of Health recommended vaccines for their age group.

## 5. Conclusions

Just over half of parents (56.7%) reported taking their children for scheduled vaccinations across the country. This might indicate that there is a substantial number of children who have not been taken for their scheduled vaccinations. Sex, age, population group, education, and locality were found to be key sociodemographic determinants of parents having taken their children for scheduled vaccinations. Parents’ experiences and views were also key determinants of parents having taken their children for scheduled vaccinations in South Africa. Initiatives that increase parents’ participation in other disease prevention interventions such as flu vaccination can increase chances of parents taking their children for scheduled vaccinations, specifically in the provinces with low proportions of parents taking children for vaccines (Free State, North West, and Limpopo). These findings are of significance especially during the time when the country is still struggling to reach a substantial proportion of its population vaccinated for COVID-19. Thus, these findings may be relevant in determining parents’ intentions to have their children receive the South African Department of Health recommended vaccines for their respective age group.

## Figures and Tables

**Table 1:** Explanatory variables.

Explanatory Variables	Final Categories
Sociodemographic variables	
Sex	Female, Male
Age group	18–29 years, 30–39 years, 40–49 years, 50–59 years, 60+ years
Population group	Black African, White, Coloured, Indian/Asian
Education level	None, Primary, Secondary, Matric, Tertiary
Employment status	Employed, Unemployed
Locality	City, Suburb, Township, Informal settlement, Rural (Traditional tribal area), Farm
Vaccine experiences	
Have you ever taken the FLU vaccine?	Yes, No
Have you ever PERSONALLY refused to take any vaccine?	Yes, No
Have you ever objected to ALLOW SOMEONE ELSE to take a vaccine?	Yes, No
Do you know anyone who has personally experienced serious side effects to any vaccine?	Yes, No
Do you know ANYONE who does not take a vaccine due to religious or cultural reasons?	Yes, No
Vaccine views	
Do you think vaccines are a good way to protect communities from disease?	Yes, No, Not sure
Do you think vaccines strengthen the immune system?	Yes, No, Not sure
When available, would you take the COVID-19 vaccine?	Yes, No, Not sure/Uncertain

**Table 2:** Characteristics of the study sample.

	Sample	%	95% CI
Total	12,708	100	
Sociodemographics			
Sex			
Female	7580	60.7	[59.8–61.6]
Male	4906	39.3	[38.4–40.2]
Population group			
Black African	9877	80.6	[79.9–81.3]
White	381	3.1	[2.8–3.4]
Coloured	1758	14.3	[13.7–15.0]
Indian/Asian	244	2.0	[1.8–2.3]
Age group (years)			
18–29	4709	40.1	[39.2–41.0]
30–39	4388	37.3	[36.5–38.2]
40–49	1788	15.2	[14.6–15.9]
50–59	618	5.3	[4.9–5.7]
60+	248	2.1	[1.9–2.4]
Educational level			
None	456	3.7	[3.4–4.0]
Primary	517	4.2	[3.8–4.5]
Secondary	3647	29.4	[28.6–30.2]
Matric	5278	42.6	[41.7–43.5]
Tertiary	2495	20.1	[19.4–20.8]
Employment status			
Employed	3848	32.3	[31.5–33.2]
Unemployed	8048	67.7	[66.8–68.5]
Locality			
City	1553	12.3	[11.8–12.9]
Suburb	1687	13.4	[12.8–14.0]
Township	5594	44.5	[43.6–45.3]
Informal settlement	1061	8.4	[8.0–8.9]
Rural (Traditional tribal area)	2380	18.9	[18.2–19.6]
Farm	305	2.4	[2.2–2.7]
Vaccine experiences			
Have you ever taken the FLU vaccine?			
Yes	3804	30.0	[29.2–30.8]
No	8887	70.0	[69.2–70.8]
Have you ever PERSONALLY refused to take any vaccine?			
Yes	1930	15.2	[14.6–15.8]
No	10,778	84.8	[84.2–85.4]
Have you ever objected to ALLOW SOMEONE ELSE to take a vaccine?			
Yes	3332	26.3	[25.5–27.0]
No	9350	73.7	[73.0–74.5]
Do you know anyone who has personally experienced serious side effects to any vaccine?			
Yes	3946	31.1	[30.3–31.9]
No	8737	68.9	[68.1–69.7]
Do you know ANYONE who does not take a vaccine due to religious or cultural reasons?			
Yes	2159	23.9	[23.1–24.8]
No	6859	76.1	[75.2–76.9]
Vaccine views			
Do you think vaccines are a good way to protect communities from disease?			
Yes	7866	62.0	[61.2–62.9]
No	841	6.6	[6.2–7.1]
Not Sure	3976	31.3	[30.5–32.2]
Do you think vaccines strengthen the immune system?			
Yes	6636	52.3	[51.5–53.2]
No	1084	8.5	[8.1–9.0]
Not Sure	4959	39.1	[38.3–40.0]
When available, would you take the COVID-19 vaccine?			
Yes	8131	72.4	[71.5–73.2]
No	1156	10.3	[9.7–10.9]
Not sure	1949	17.3	[16.7–18.1]

CI = Confidence Interval. Subtotals are not always equal to the overall total due to nonresponse or missing data.

**Table 3:** Parents’ taking children for scheduled vaccination by sociodemographics, experiences, and views.

	Sample	%	95% CI	*p* Value
Total	12,708	56.7	[55.1–58.2]	
Sociodemographics				
Sex				
Female	7580	49.6	[47.6–51.6]	<0.001
Male	4906	35.9	[33.6–38.3]	
Population group				
Black African	9877	37.8	[36.2–39.4]	<0.001
White	381	69.7	[61.2–77.0]	
Coloured	1758	51.5	[48.3–54.6]	
Indian/Asian	244	69.5	[61.1–76.8]	
Age group				
18–29	4709	37.2	[35.7–38.6]	<0.001
30–39	4388	39.4	[37.9–41.0]	
40–49	1788	43	[40.6–45.6]	
50–59	618	47.2	[42.8–51.5]	
60+	248	55.1	[47.6–62.5]	
Educational level				
None	456	43.3	[33.7–53.4]	0.007
Primary	517	41.9	[33.7–50.6]	
Secondary	3647	38.7	[36.1–41.3]	
Matric	5278	43.4	[41.2–45.8]	
Tertiary	2495	49.1	[45.4–52.7]	
Employment status				
Employed	3848	45.6	[43.2–48.0]	<0.001
Unemployed	8048	40.2	[38.4–42.0]	
Locality				
City	1553	43.3	[38.7–48.0]	<0.001
Suburb	1687	58	[53.1–62.7]	
Township	5594	41.9	[39.9–44.0]	
Informal settlement	1061	37.9	[32.7–43.4]	
Rural (Traditional tribal area)	2380	34.8	[31.5–38.2]	
Farm	305	52.8	[41.7–63.5]	
Vaccine experiences				
Have you ever taken the FLU vaccine?				
Yes	3804	62.1	[59.0–65.0]	<0.001
No	8887	34.3	[32.5–36.1]	
Have you ever PERSONALLY refused to take any vaccine?				
Yes	1930	52.3	[48.3–56.3]	<0.001
No	10,778	41.8	[40.1–43.5]	
Have you ever objected to ALLOW SOMEONE ELSE to take a vaccine?				
Yes	3332	40.6	[37.7–43.6]	0.053
No	9350	44	[42.2–45.9]	
Do you know anyone who has personally experienced serious side effects to any vaccine?				
Yes	3946	47.9	[45.1–50.6]	<0.001
No	8737	41.6	[39.7–43.5]	
Do you know ANYONE who does not take a vaccine due to religious or cultural reasons?				
Yes	2159	50.5	[46.7–54.3]	0.004
No	6859	44	[41.9–46.2]	
Vaccine views				
Do you think vaccines are a good way to protect communities from disease?				
Yes	7866	47.2	[45.1–49.2]	<0.001
No	841	29.6	[24.8–35.0]	
Not Sure	3976	37.2	[34.6–39.9]	
Do you think vaccines strengthen the immune system?				
Yes	6636	48.4	[46.2–50.5]	<0.001
No	1084	38.8	[32.0–46.1]	
Not Sure	4959	36.2	[34.0–38.6]	
When available, would you take the COVID-19 vaccine?				
Yes	8131	44.4	[42.4–46.4]	0.604
No	1156	41.9	[37.0–46.9]	
Not sure	1949	44.3	[40.9–47.7]	

CI = Confidence Interval. Subtotals are not always equal to the overall total due to nonresponse or missing data.

**Table 4:** Multivariate logistic regression of factors that influence parents’ having taken their children for scheduled vaccinations.

	aOR	[95% CI]	*p* Value
Sex			
Female (Ref)			
Male	0.53	[0.45–0.61]	<0.001
Population group			
Black African (Ref)			
White	4.08	[2.79–5.96]	<0.001
Coloured	1.72	[1.42–2.08]	<0.001
Indian/Asian	3.50	[2.23–5.49]	<0.001
Age group			
18–29 (Ref)			
30–39	1.19	[1.05–1.35]	0.007
40–49	1.27	[1.08–1.49]	0.004
50–59	1.29	[1.02–1.64]	0.035
60+	1.90	[1.24–2.90]	0.003
Employment status			
Employed (Ref)			
Unemployed	0.98	[0.84–1.14]	0.781
Locality			
City (Ref)			
Suburb	1.78	[1.33–2.38]	<0.001
Township	1.41	[1.09–1.82]	0.009
Informal settlement	1.45	[1.04–2.03]	0.029
Rural (Traditional tribal area)	0.96	[0.72–1.28]	0.772
Farm	1.15	[0.69–1.90]	0.593
Have you ever taken the FLU vaccine?			
Yes (Ref)			
No	0.33	[0.28–039]	<0.001
Have you ever PERSONALLY refused to take any vaccine?			
Yes (Ref)			
No	0.81	[0.64–1.01]	0.057
Do you know anyone who has personally experienced serious side effects to any vaccine?			
Yes (Ref)			
No	0.77	[0.66–0.90]	0.001
Do you know ANYONE who does not take a vaccine due to religious or cultural reasons?			
Yes (Ref)			
No	0.86	[0.72–1.01]	0.073
Do you think vaccines are a good way to protect communities from disease?			
Yes (Ref)			
No	0.50	[0.33–0.77]	0.001
Not Sure	0.87	[0.71–1.06]	0.173
Do you think vaccines strengthen the immune system?			
Yes (Ref)			
No	0.76	[0.53–10.8]	0.123
Not Sure	0.68	[0.56–0.82]	<0.001

CI = Confidence Interval. aOR = Adjusted Odds Ratio.

## Data Availability

Available on request from corresponding author upon reasonable time.
